# Elusive roles for reactive astrocytes in neurodegenerative diseases

**DOI:** 10.3389/fncel.2015.00278

**Published:** 2015-08-03

**Authors:** Lucile Ben Haim, Maria-Angeles Carrillo-de Sauvage, Kelly Ceyzériat, Carole Escartin

**Affiliations:** ^1^Commissariat à l'Energie Atomique et aux Energies Alternatives, Département des Sciences du Vivant, Institut d'Imagerie Biomédicale, MIRCenFontenay-aux-Roses, France; ^2^Neurodegenerative Diseases Laboratory, Centre National de la Recherche Scientifique, Université Paris-Sud, UMR 9199Fontenay-aux-Roses, France

**Keywords:** astrocyte reactivity, neuron-astrocyte interactions, neuroinflammation, Alzheimer's disease, Huntington's disease, amyotrophic lateral sclerosis, Parkinson's disease

## Abstract

Astrocytes play crucial roles in the brain and are involved in the neuroinflammatory response. They become reactive in response to virtually all pathological situations in the brain such as axotomy, ischemia, infection, and neurodegenerative diseases (ND). Astrocyte reactivity was originally characterized by morphological changes (hypertrophy, remodeling of processes) and the overexpression of the intermediate filament glial fibrillary acidic protein (GFAP). However, it is unclear how the normal supportive functions of astrocytes are altered by their reactive state. In ND, in which neuronal dysfunction and astrocyte reactivity take place over several years or decades, the issue is even more complex and highly debated, with several conflicting reports published recently. In this review, we discuss studies addressing the contribution of reactive astrocytes to ND. We describe the molecular triggers leading to astrocyte reactivity during ND, examine how some key astrocyte functions may be enhanced or altered during the disease process, and discuss how astrocyte reactivity may globally affect ND progression. Finally we will consider the anticipated developments in this important field. With this review, we aim to show that the detailed study of reactive astrocytes may open new perspectives for ND.

## Introduction

Astrocytes become reactive in response to virtually all pathological conditions in the central nervous system (CNS), both following acute injuries (stroke, trauma) and during progressive diseases (tumors, epilepsy and ND see Box 1 and Table 1). Astrocyte reactivity is observed in many mammalian and bird species. In non-mammalian species, which have low number of or no parenchymal astrocytes, it is unclear whether *bona fide* astrocyte reactivity exists (Appel, 2013). Yet, in lampreys, newts and frogs, astrocyte-like cells react to injury and form a glial bridge promoting axonal regeneration (Bloom, 2014). In Drosophila, glial cells with some typical astrocyte functions display strong phagocytic activity and morphological changes following neuronal degeneration (Freeman, 2015).

**Table 1:** **Reactive astrocytes are found in vulnerable brain regions in animal models and patients with ND**.

		**When**	**Where**	**References**	**Comments**
**AD**	Patients	Before clinical symptoms. GFAP levels increase with Braak stage	Entorhinal cortex and hippocampus. Gradual progression to temporal, frontal and parietal lobes	Simpson et al., 2010; Carter et al., 2012	
	Murine models	May start before amyloid deposition. Prominent when plaques are formed	Primarily around amyloid plaques. (Brain region depends on the model)	Heneka et al., 2005; Duyckaerts et al., 2008; Olabarria et al., 2010	Astrocytes located far from plaques are atrophied in 3xTg-AD mice
**HD**	Patients	Already visible at grade 0 in putamen.	Primarily in caudate and putamen. Later in motor cortex, globus pallidus, thalamus, hippocampus	Vonsattel et al., 1985; Faideau et al., 2010	
	Murine models	Late or no reactivity	Striatum	Tong et al., 2014; Ben Haim et al., 2015	Strong reactivity in models with neuronal death (Lenti-Htt82Q or toxins)
**ALS**	Patients	Before motor symptoms	Ventral and dorsal horns in the spinal cord. Lateral descending corticospinal tracts, subcortical white matter, cortical gray matter in the brain	Maragakis and Rothstein, 2006; Philips and Robberecht, 2011	
	Murine models	Before motor symptoms	Pattern similar to that in patients	Hall et al., 1998; Barbeito et al., 2004; Maragakis and Rothstein, 2006	
**PD**	Patients	Follows dopaminergic cell death	*Substantia nigra*, correlates with the severity of neuronal loss	Forno et al., 1992; Damier et al., 1993	
	MPTP- monkeys	Follows dopaminergic cell death	*Substantia nigra*	Barcia et al., 2004	
	MPTP-mice 6-OHDA rats	Follows microglial activation, peaks at 4–5 days after intoxication	*Substantia nigra* and striatum	Sheng et al., 1993; Kohutnicka et al., 1998; Hirsch and Hunot, 2009

Box 1 **Terminology and definitions**

**“*Neuroinflammation”*** defines the state of reactivity of astrocytes and microglia induced by pathological conditions. It may be associated with the recruitment of peripheral macrophages and lymphocytes. Reactive astrocytes and microglia mediate the innate immune responses in the brain (Heneka et al., 2014).

In this review, the term “***astrogliosis”*** will not be used because it implies the notion of astrocyte proliferation. In fact, in most injury or disease models, astrocytes do not proliferate (see Section Do Reactive Astrocytes Proliferate in ND?) and thus, reactive astrogliosis is a confounding term.

“***Astrocyte reactivity***” or “***reactive astrocytes”*** refer to astrocytes that respond to any pathological condition in the CNS. Astrocytes are considered reactive when they become hypertrophic and overexpress the intermediate filament GFAP. This “minimal definition” of reactive astrocytes is thus based on the two most universal hallmarks of reactivity, but this does not exclude that additional transcriptional, morphological and functional changes occur in a disease-specific manner, as discussed later in this review. Astrocyte reactivity involves the activation of a transcriptional program triggered by specific signaling cascades (see Section How Do Astrocytes Become Reactive?) that results in long-lasting changes in morphology and function, persisting over several hours, days or even decades. This should be distinguished from **“activated astrocytes,”** which are stimulated by exposure to neurotransmitters, for example. This transient response involves intracellular Ca^2+^ on the millisecond to second time scale, and is sometimes accompanied by subtle morphological changes (Bernardinelli et al., 2014), but not long term increases in GFAP gene expression or morphological hypertrophy.

“***Glial scar***” is a specific form of astrocyte reactivity, which is irreversible and involves major morphological remodeling of reactive astrocytes along the disrupted parenchyma.

**“*Resting astrocytes”*** will be used to denote astrocytes that are not reactive. However, it does not mean that astrocytes are inactive; instead, it refers to a non-disease state, or a “homeostically active” state.

Astrocyte reactivity involves morphological, transcriptional and functional changes that we will try to cover in this review. For the sake of clarity, we will focus primarily on Alzheimer's (AD) and Huntington's diseases (HD), as well as amyotrophic lateral sclerosis (ALS) and Parkinson's disease (PD). In particular, we aim to illustrate that astrocyte reactivity is a shared and central feature in ND that requires further characterization.

It has been very difficult to distinguish the contribution of astrocytes from that of microglia because they usually become reactive in concert and both are involved in neuroinflammation (see definitions in Box 1). However, they have quite different functions in the brain in normal conditions; therefore, they may also play different roles during ND. Cell-type specific approaches based on viral vectors or transgenesis offer a unique opportunity to understand the roles of reactive astrocytes (Davila et al., 2013). In this review, we will focus on reactive astrocytes in ND. Excellent reviews recently published on microglia in ND can be found elsewhere (Hanisch and Kettenmann, 2007; Heneka et al., 2014).

## Reactive astrocytes in ND: definitions and general considerations

### A brief history

In 1856, Rudolf Virchow first described “neuroglia” as a connective tissue with embedded nerve cells (Virchow, 1856). The development of microscopic and histological techniques by Camilo Golgi, Santiago Ramón y Cajal and Pio del Rio Hortega later revealed the morphology of astrocytes and their extraordinary diversity (Somjen, 1988; Kettenmann and Ransom, 2004). The first description of astrocyte reactivity also dates from the nineteenth century, when Virchow reported that the spinal cord tissue was more fibrillar in neurosyphilis patients than in healthy individuals (Weigert, 1895; Oberheim et al., 2008). The concept of astrocyte reactivity truly emerged with the discovery of the intermediate filament (IF) protein GFAP (Eng et al., 1971) and the development of immunohistological staining for this protein (Eng et al., 2000). Strong GFAP expression in astrocytes became the hallmark of reactivity (Bignami and Dahl, 1976), even though other IF such as vimentin and nestin are also upregulated by reactive astrocytes.

### Morphological changes

Another cardinal feature of astrocyte reactivity is hypertrophy, which was reported by early neuropathologists. Reactive astrocytes display an enlarged cell body and processes (Wilhelmsson et al., 2006). In addition, astrocyte arborization is reorganized with reactivity: the number of primary processes changes (Wilhelmsson et al., 2006) or they polarize toward the site of injury (Bardehle et al., 2013) or toward amyloid plaques in AD (see below).

Less is known about the thin distal processes in astrocytes called perisynaptic processes (PAP), which contact synapses. PAP are dynamic and they influence synaptic transmission in physiological conditions (Oliet et al., 2001; Genoud et al., 2006; Bernardinelli et al., 2014). It is experimentally challenging to monitor morphological changes in PAP that are smaller than the diffraction limit. New microscopy techniques will allow the study of PAP with higher resolution in both resting and reactive astrocytes (Panatier et al., 2014).

Astrocytes occupy separate and non-overlapping spatial domains (Bushong et al., 2002). This organization seems fairly insensitive to reactivity during ND because an increase in domain overlap occurs only after severe insults such as recurring epilepsy, but not in AD models (Oberheim et al., 2008).

### Reactive astrocytes in commonly studied ND

AD is the most common form of dementia, characterized by cognitive deficits including learning impairment and memory loss (Querfurth and Laferla, 2010). The brains of AD patients display extracellular amyloid depositions composed of amyloid β (Aβ) peptides and intracellular neurofibrillary tangles formed by hyperphosphorylated Tau protein. AD is characterized by severe neuronal loss; primarily located in the hippocampus and the entorhinal cortex (Querfurth and Laferla, 2010). More than 100 transgenic mouse models of AD are now available (Duyckaerts et al., 2008, see also www.alzforum.org). Most involve the expression of mutated amyloid precursor protein (APP), presenilin 1 (PS1), PS2 and/or Tau, and they replicate some neuropathological features and functional alterations of AD as well as memory deficits (Gotz and Ittner, 2008). Astrocyte reactivity can be detected in the brain of AD patients with imaging and proteomic techniques before the onset of symptoms (Owen et al., 2009; Carter et al., 2012). Similarly, foci of reactive astrocytes are detected at early stages in some mouse models, even before amyloid deposition (Heneka et al., 2005). Reactive astrocytes are usually found around amyloid plaques (Nagele et al., 2003; Wyss-Coray et al., 2003, Table 1). However, plaques can also be devoid of reactive astrocytes and patches of reactive astrocytes may be found in the absence of plaques in patients (Simpson et al., 2010). In addition, atrophied astrocytes may be located at a distance from plaques in some mouse models (Olabarria et al., 2010, see Tables 1, 2).

**Table 2:** **Main behavioral, cellular, and molecular features of several mouse models of ND**.

**Disease modeled**	**Model**	**Species**	**Genetic construct**	**Promoter**	**Lifespan**	**Major symptoms**	**Histopathological features**	**Vulnerable brain regions**	**Original references**
**AD**	APP/PS1dE9	Mouse	Mo/hu APP_Swe_, Hu PSEN1 deltaE9	MoPrP	Normal	Spatial memory impairment Reduced LTP	Extracellular Aβ depositions No extensive neuronal death	Hippocampus Cortex	Jankowsky et al., 2004
	3xTg-AD	Mouse	HuAPP_Swe_, Mo PSEN1^M146V^, Hu MAPT^P301L^,	MoThy1.2 (for APP and MAPT); endogenous moPSEN1 promoter (for PSEN1)	Normal	Spatial memory impairment Reduced basal synaptic transmission and LTP	Extracellular Aβ depositions Tau pathology in CA1 neurons No extensive neuronal death	Subiculum Hippocampus Entorhinal cortex	Oddo et al., 2003
	Tg2576	Mouse	HuAPP_Swe_	Hamster PrP	Normal	Spatial memory impairment Reduced LTP	Extracellular Aβ depositions No extensive neuronal death	Hippocampus Frontal, entorhinal and occipital cortex	Hsiao et al., 1996
	APPPS1	Mouse	Mo/hu APP_Swe_; Hu PSEN1^L166P^	Mo Thy1	Normal	Spatial memory impairment Reduced LTP	Extracellular Aβ depositions Local neuronal death in the dentate gyrus Altered dendritic spine morphology	Hippocampus Cortex	Radde et al., 2006
**HD**	R6/2	Mouse	Exon 1 of huHtt with 150 CAG	1 kb huHtt	10–13 weeks	Altered motor coordination and gait Involuntary movements Seizures Cognitive deficits (selective discrimination learning, contextual fear conditioning) Alteration of MSN electrophysiology	NII Striatal atrophy No extensive neuronal death Alteration of MSN morphology	Striatum Motor cortex	Mangiarini et al., 1997
	Hdh140	Mouse	Knock-in of Mo/hu exon 1 Htt with 140 CAG	Endogenous moHtt promoter	Normal	Altered motor coordination Alteration of MSN electrophysiology	NII Striatal atrophy Low expression of neuronal transcripts	Striatum Motor cortex	Menalled et al., 2003
	zQ175	Mouse	Knock-in of Mo/hu exon 1 Htt with 175 CAG (derived from Hdh140)	Endogenous moHtt promoter	Normal	Altered motor coordination Cognitive deficits (selective discrimination learning)	NII Striatal atrophy Low expression of neuronal transcripts	Striatum Motor cortex	Menalled et al., 2012
	BACHD	Mouse	Full length huHtt with 97 CAG/CAA	HuHtt	Normal	Altered motor coordination Alteration of MSN electrophysiology	Extranuclear aggregates Striatal and cortical atrophy No extensive neuronal death	Striatum Motor cortex	Gray et al., 2008
	Lenti-Htt82Q(lentiviral-based)	Mouse	Lentiviral vector encoding 171 first amino acids of huHtt with 82 Q	PGK	Normal	Behavioral alterations not tested in mice	Cytoplasmic and nuclear inclusions MSN loss	Striatum	De Almeida et al., 2002
ALS	SOD1G93A	Mouse	HuSOD1^G93A^	HuSOD1	5 months	Weight loss Hindlimb weakness Progressive paralysis	Loss of ventral SC motor neurons Degeneration of ventral root axons SOD1 inclusions in the SC and brain	Spinal cord Motor cortex	Gurney et al., 1994
	SOD1G93A	Rat	HuSOD1^G93A^	HuSOD1	3–4 months	Abnormal gait Quick hindlimb paralysis Weight loss Muscle atrophy and denervation	Loss of ventral SC motor neurons Degeneration of ventral root axons Vacuolar degeneration SOD1 inclusions in the SC and brain	Spinal cord Brain	Howland et al., 2002
PD	MPTP	Mouse	N/A	N/A	N/A	Altered rearing and coordination	Loss of DA neurons Reduced dopamine levels instriatum	SNpc Striatum	Heikkila et al., 1984
	MPTP	Non-human primate	N/A	N/A	N/A	Bradykinesia, rigidity, tremor	Loss of DA neurons Reduced dopamine levels incaudate-putamen	SNpc Caudate putamen	Burns et al., 1983

*Common mouse models of ND were included in the table if they are mentioned at least twice in the text. APPSwe, KM670/671NL (Swedish mutation); DA, dopaminergic. Hu, human; Mo, mouse; MSN, medium-sized spiny neurons; NII, neuronal intranuclear inclusions; PGK, phosphoglycerate kinase; PrP, Prion Protein; Q, glutamine, SNpc, Substantia nigra pars reticulata*.

HD is a fatal genetic ND caused by an autosomal dominant mutation, involving the expansion of glutamine (Q) repeats in the protein huntingtin (Htt). HD patients present psychiatric, cognitive and motor symptoms, the most characteristic being progressive chorea (Vonsattel et al., 1985). HD is characterized by the extensive loss of GABAergic neurons in the caudate and putamen (striatum) and by mutant Htt (mHtt) aggregates. Many HD transgenic mouse models exist, which were generated by expressing mHtt under the endogenous Htt promoter (knock-in) or using various transgenic constructs (see Table 2). Astrocyte reactivity is an early feature of HD because GFAP immunoreactivity is detected in the striatum of presymptomatic carriers and it increases with disease progression (Faideau et al., 2010). Strikingly, no clear evidence of astrocyte reactivity exists in most HD models (Tong et al., 2014; Ben Haim et al., 2015, Table 1). Instead, HD astrocytes show functional alterations (see Section What Do Reactive Astrocytes Do or Fail to Do During ND?) in the absence of the main features of reactivity (hypertrophy and high GFAP expression).

ALS is characterized by the progressive loss of upper motor neurons in the motor cortex and lower motor neurons in the spinal cord and brainstem, resulting in progressive muscle atrophy, weakness and spasticity (Rothstein et al., 1992). Murine models overexpressing mutated forms of superoxide dismutase 1 (mSOD1), identified in familial forms of ALS, develop progressive motor neurodegeneration that mimics the pathogenic features of ALS (Turner and Talbot, 2008, Table 2). More recently, new genetic loci associated with familial ALS have been identified, like the *43 kDa transactivation-response DNA-binding protein (TDP-43)*, and new mouse models are being developed (Robberecht and Philips, 2013). Reactive astrocytes are observed in both ALS patients and models (Table 1). They appear in vulnerable regions and the degree of reactivity correlates with the level of neurodegeneration (Barbeito et al., 2004).

PD is characterized by the loss of dopaminergic neurons in the substantia nigra (SN), resulting in dopamine deficiency in the striatum and alteration of the basal ganglia circuitry. This causes major motor symptoms, such as akinesia, bradykinesia, tremor, rigidity and postural instability (Agid, 1991), as well as non-motor alterations such as cognitive fluctuations (Witjas et al., 2002). The first animal models of PD were based on toxins that specifically induce the degeneration of dopaminergic neurons in the SN pars compacta (SNpc) in rodents or primates (e.g., 6-hydroxydopamine [6-OHDA], 1-methyl-4-phenyl-1,2,3,6-tetrahydropyridine [MPTP] or rotenone). Transgenic mice harboring genes mutated in familial PD (α-synuclein, leucine-rich repeat kinase 2…) were subsequently developed (Beal, 2010, see Table 2). The involvement of microglial cells in PD has been more extensively studied than that of astrocytes. Yet, astrocyte reactivity is detected in the SNpc of patients with PD, individuals intoxicated with MPTP and in animal models (Forno et al., 1992; Hirsch and Hunot, 2009, Table 1).

### Do reactive astrocytes proliferate in ND?

The original definition of astrocyte reactivity included the notion of proliferation. The idea that reactive astrocytes proliferate is based on the misleading observation that the number of GFAP^+^ cells increases after injury (Dimou and Gotz, 2014). Most astrocytes in the adult mouse CNS express GFAP at undetectable levels under physiological conditions. Upon injury or disease, reactive astrocytes upregulate GFAP, leading to an increased number of GFAP^+^ cells. Recent evidence based on BrdU incorporation or Ki67 labeling reveals that astrocyte proliferation is very limited, especially in ND. The exact value depends on the model, age and detection method. For example, reactive astrocytes do not proliferate in the APP/PS1dE9 mouse model of AD (Kamphuis et al., 2012), and represent less than 3% of total proliferating cells in the APPPS1 mouse model of AD (Sirko et al., 2013) and less than 7% in a model of ALS (Lepore et al., 2008a). Proliferating astrocytes account for only 1% of total gray matter astrocytes in APPPS1 mice (Sirko et al., 2013). In the temporal cortex of AD patients, GFAP^+^ cells were carefully quantified by co-labeling with ubiquitous astrocyte markers (glutamine synthase [GS] or aldehyde dehydrogenase 1 family, member L1). This analysis confirms that the high density of GFAP^+^ cells is explained by enhanced GFAP expression and cortical atrophy (Serrano-Pozo et al., 2013).

### Can astrocyte reactivity be reproduced *in vitro*?

With the development of *in vitro* systems to study astrocytes (McCarthy and De Vellis, 1980), it became possible to study reactive astrocytes in a dish. Human astrocytes can also be grown *in vitro*, either from fetuses or biopsies (Sharif and Prevot, 2012) or generated from induced pluripotent stem cells, including from patients (Krencik and Ullian, 2013). Generally, primary astrocytes are exposed to cytokines such as interleukins (IL), tumor necrosis factor alpha (TNFα) and interferon gamma (IFNγ), which induce many transcriptional and functional changes (Sofroniew, 2009; Sofroniew and Vinters, 2010). Describing them all is beyond the scope of this review. The main limitation to *in vitro* studies is that astrocytes in a dish show signs of reactivity, even in the absence of stimulus. They express high levels of GFAP and usually have a flat, polygonal morphology, very different from the bushy morphology observed *in situ*. This precludes the identification of the hallmarks of astrocyte reactivity. Co-culture with neurons triggers a stellate morphology and low GFAP expression, suggesting that neurons release factors that maintain astrocytes in a resting state (see Section The Molecular Triggers of Reactivity). More recently, new methods have been developed to reduce astrocyte reactivity *in vitro*, such as exposure to heparin-binding EGF-like growth factor (Foo et al., 2011) or 3D polymer matrix (Puschmann et al., 2013). Given the above-mentioned limitations, we will focus on results obtained in animal models, or even more relevant, in patient brains.

## How do astrocytes become reactive?

### The molecular triggers of reactivity

Astrocyte reactivity is triggered by any alteration in brain homeostasis. Astrocytes are equipped with many receptors and intracellular signaling cascades to respond quickly to changes in their environment (Buffo et al., 2010; Burda and Sofroniew, 2014). They express many receptors, including pattern recognition receptors (PRR), that detect abnormal signals in the extracellular space (viral or bacterial molecules, serum proteins, aggregated proteins such as Aβ…), increased concentrations of some molecules (cytokines, chemokines, purines) and even the absence of “normal” signals from neighboring cells (growth factors, neurotransmitters…) (Buffo et al., 2010; Burda and Sofroniew, 2014; Kigerl et al., 2014). Indeed, astrocytes, like microglia, seem to be actively maintained in a resting state. For instance, knocking out fibroblast growth factor (FGF) receptors or β1 integrin (a subunit of the integrin receptor family that binds extracellular matrix molecules) in astrocytes, results in astrocyte reactivity in absence of pathological stimuli (Robel et al., 2009; Kang et al., 2014).

Extracellular molecules inducing astrocyte reactivity have primarily been studied in acute injury models involving scar formation (Burda and Sofroniew, 2014). Such acute lesions involve the breach of the blood-brain-barrier (BBB) and infiltration of immune cells. By contrast, these events occur very progressively in ND, if ever (Zlokovic, 2008). Therefore, although some molecular triggers are shared between acute injuries and ND, molecules such as endothelins or serum proteins are probably not involved in the initiation of astrocyte reactivity in ND.

The exact molecular triggers that occur during the initial stages of ND, before significant neurodegeneration takes place, are unknown. It is probable that glial cells (both astrocytes and microglial cells) can detect even mild neuronal dysfunction (altered neurotransmission, release of stress signals, and abnormally folded proteins). Indeed, astrocytes are very well positioned at the tripartite synapse to detect abnormal synaptic activity and microglial cells permanently monitor the brain parenchyma. Once activated by such signals, glial cells further release active molecules to set up a reactive state. For example, purines, pro-inflammatory cytokines and growth factors may be released by reactive astrocytes and, in even larger amounts by activated microglia (Buffo et al., 2010, see Section Release of Active Molecules).

**Figure 1:**
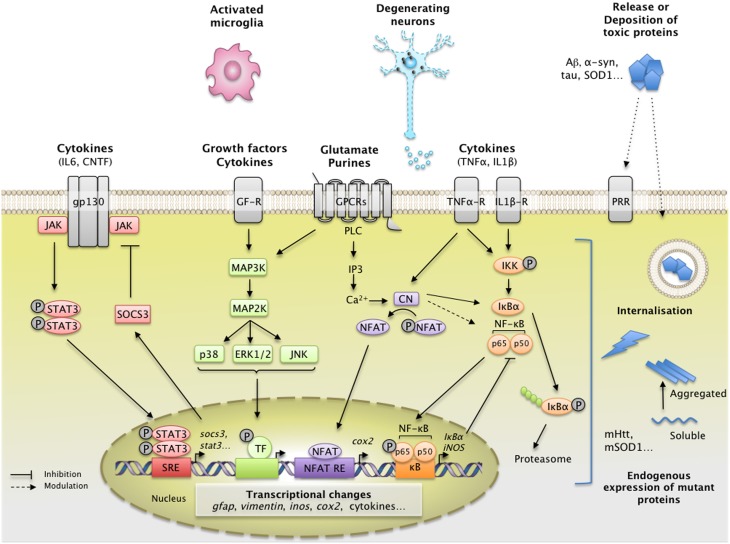
**Main extracellular stimuli and intracellular signaling pathways leading to astrocyte reactivity in ND**. Dysfunctional neurons, activated microglia, and astrocytes themselves release a wide range of molecules, which bind specific receptors at the astrocyte plasma membrane. These signals activate intracellular pathways such as the JAK/STAT3 pathway (in red), the NF-κB pathway (in orange), the CN/NFAT pathway (in purple), or the MAPK pathway (in green). **The JAK/STAT3 pathway** is activated by interleukins such as IL-6 or CNTF. Upon cytokine binding, the kinase JAK is activated and STAT3 is recruited to the gp-130 receptor. JAK phosphorylates STAT3, which dimerizes and translocates to the nucleus, where it binds consensus sequences (STAT responsive element, SRE) in the promoter region of its target genes. In astrocytes, the JAK/STAT3 pathway regulates the transcription of *gfap, vimentin*, and *stat3* itself. STAT3 also induces the expression of SOCS3, the endogenous inhibitor of the JAK/STAT3 pathway, which mediates an inhibitory feedback loop. **The NF-κB pathway** is activated by pro-inflammatory cytokines such as TNFα and IL-1β. The canonical NF-κB pathway involves the activation of the IKK complex by receptor-bound protein kinases, leading to the phosphorylation of IκBα, the master inhibitor of NF-κB. Upon phosphorylation, IκBα is polyubiquinated and targeted to the proteasome for degradation. The NF-κB subunits p50 and p65 then translocate to the nucleus, where they activate the transcription of various target genes such as inducible nitric oxide synthase and *cox2*. Like the JAK/STAT3 pathway, NF-κB induces the transcription of its own inhibitor, IκBα. **The CN/NFAT pathway** is activated by cytokines such as TNFα or by glutamate. CN is a Ca^2+^-dependent phosphatase with many regulatory effects on the NF-κB pathway depending on the initial trigger and cellular context. CN also activates NFAT by dephosphorylation. NFAT binds specific promoter sequences and activates the expression of target genes (*cox2*). **The MAPK pathway** is activated by growth factors and cytokines which initiate a phosphorylation cascade. Upon activation, ERK1/2, p38 and c-jun also regulate gene transcription through the activation of a specific set of transcription factors (TF). ND are characterized by intracellular and/or extracellular depositions of pathologic proteins (such as Aβ, Tau, and mHtt, which are shown in blue). In ND, pathological proteins can either be endogenously expressed or internalized by astrocytes. They represent “danger associated molecular patterns” that bind specific pattern recognition receptors (PRR) at the membrane or within astrocytes. These abnormal proteins can interfere with intracellular signaling pathways, activating or inhibiting various signaling proteins (represented as lightning, see Section Additional levels of Complexity and **Figure 3**). The precise molecular mechanisms involved in astrocytes are mostly unknown. These complex signaling cascades strongly affect the astrocyte transcriptome and lead to astrocyte reactivity. Abbreviations: NFAT RE, NFAT responsive element; GF-R, Growth factor receptor; GPCR, G-protein coupled receptor.

Importantly, in ND, mutant proteins (e.g., mHtt, mSOD1) may be directly expressed by astrocytes or toxic proteins (Aβ, hyperphosphorylated tau, α-synuclein) can be taken up by astrocytes and activate them (see Figure 1). Lentiviral-mediated expression of mHtt specifically in striatal astrocytes increases GFAP expression and induces cellular hypertrophy (Faideau et al., 2010). Similarly, the expression of SOD1^G86R^, α-synuclein^A53T^, or tau (either WT or P301L mutant) in astrocytes, induces their reactivity (Gong et al., 2000; Dabir et al., 2006; Gu et al., 2010). The precise molecular mechanisms linking the accumulation of intracellular toxic proteins in astrocytes to reactivity remain to be characterized (see Figure 1). Cytosolic PRR that can detect intracellular “danger associated molecular patterns” have been described in microglial cells, but much less is known about their role in astrocytes (Heneka et al., 2014).

Other molecular triggers of reactivity (e.g., cytokines, growth factors, purines), bind to their cognate receptors at the astrocyte membrane and activate various intracellular signaling cascades. These include the Janus Kinase/Signal Transducer and Activator of Transcription (JAK/STAT) pathway, the Nuclear Factor of Kappa light polypeptide gene enhancer in B-cells (NF-κB) pathway, the calcineurin (CN) pathways and the Mitogen-Activated Protein Kinase (MAPK) pathway (Figure 1).

### The JAK/STAT3 pathway

The JAK/STAT3 pathway is a ubiquitous cascade that predominantly mediates cytokine signaling in cells. It regulates the expression of genes involved in many functions including cell growth, proliferation, differentiation and inflammation. Cytokines of the interleukin family (e.g., IL-6, ciliary neurotrophic factor [CNTF] and leukemia inhibitory factor) signal through specific cell-surface receptors possessing the glycoprotein 130 receptor (gp130) subunit. Upon binding, they trigger the assembly of multimeric receptors, leading to the phosphorylation and nuclear translocation of STAT3, and the transcription of its target genes (Levy and Darnell, 2002 and see Figure 1 for a detailed description of the pathway). Interestingly, the JAK/STAT3 pathway also controls the onset of astrogliogenesis during brain development (He et al., 2005), by promoting the expression of mature astrocyte genes such as *gfap* and *S100*β (Kanski et al., 2014).

**Figure 2:**
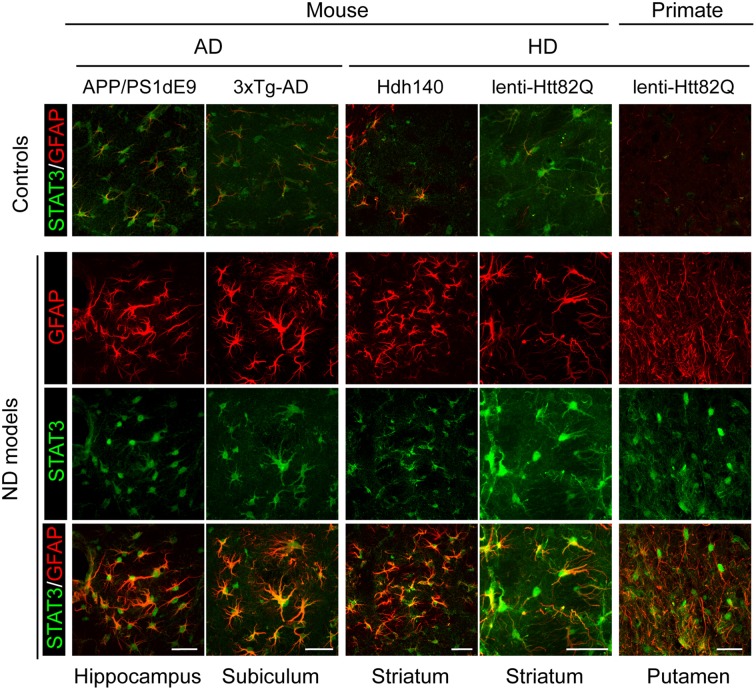
**Activation of the JAK/STAT3 pathway is a common feature of astrocyte reactivity in various ND models**. Images of brain sections from several models of AD and HD (APP/PS1dE9 mice, 3xTg-AD mice, Hdh140 mice, and the murine and primate lenti-Htt82Q-based models of HD). For all ND models, STAT3 (green) accumulates in the nucleus of reactive astrocytes labeled with GFAP (red) in specific vulnerable regions (as indicated at the bottom). The first line shows the merged STAT3 (green)/GFAP (red) staining in age-matched control animals for each model. Scale bars: 20 μm (mouse) and 40 μm (primate). Adapted from Ben Haim et al. (2015).

It is well established that the JAK/STAT3 pathway mediates astrocyte reactivity and scar formation in models of acute injuries (Okada et al., 2006; Herrmann et al., 2008). Fewer studies have been performed in ND models. Phospho-STAT3 (pSTAT3) is detected in the nucleus of reactive astrocytes (as well as microglia and motor neurons) in the spinal cord of mouse models and patients with ALS (Shibata et al., 2009, 2010). STAT3 accumulates in the nucleus of reactive astrocytes in the hippocampus of transgenic mouse models of AD and in the striatum of murine and primate models of HD (Ben Haim et al., 2015). Activation of the STAT3 pathway seems to be a universal feature of astrocyte reactivity in ND models, shared between disease models, brain regions and animal species (Figure 2). Pharmacological inhibition of JAK2 in the MPTP mouse model of PD significantly decreases pSTAT3 and GFAP levels, suggesting that the JAK/STAT3 pathway is required to induce astrocyte reactivity (Sriram et al., 2004). However, this pathway is active in all brain cells; therefore, non-specific effects of JAK2 inhibitors on other cell types cannot be ruled out. To overcome this limitation, we used lentiviral vectors to overexpress suppressor of cytokine signaling 3 (SOCS3), the endogenous inhibitor of the JAK/STAT3 pathway, selectively in astrocytes of the adult mouse brain. SOCS3 overexpression prevented the nuclear accumulation of STAT3 and GFAP upregulation in mouse models of ND. Furthermore, SOCS3-expressing astrocytes displayed a resting morphology, showing that the JAK/STAT3 pathway is responsible for astrocyte reactivity in these models (Ben Haim et al., 2015). Interestingly, a recent paper showed that the *Drosophila* ortholog of STAT3 also modulates the reactivity of glial cells following injury (Doherty et al., 2014). Therefore, the JAK/STAT3 pathway is a conserved and central pathway for astrocyte reactivity.

### The NF-κB pathway

The NF-κB pathway is another pathway associated with neuroinflammation. It is involved in many cellular processes including immune responses, inflammation, cell division and apoptosis (Mattson and Meffert, 2006 see Figure 1 for a detailed description of the pathway). This pathway is activated by several known pro-inflammatory agents (e.g., lipopolysaccharide [LPS], IL-1β, TNFα) (Kaltschmidt et al., 2005). The NF-κB pathway is found activated during ND. Following microinjection of Aβ1-42 oligomers into the rat cortex, NF-κB activation is detected in some GFAP^+^ astrocytes, along with cyclooxygenase 2 (COX2) and IL-1β, two NF-κB target genes (Carrero et al., 2012). NF-κB accumulates in astrocyte nuclei in the R6/2 model of HD (Hsiao et al., 2013) and in the spinal cord of ALS patients (Migheli et al., 1997). However, experiments involving an NF-κB-GFP reporter construct in ALS mice demonstrate that this pathway is predominantly active in microglial cells in the spinal cord (Frakes et al., 2014). In fact, the NF-κB pathway seems to be active in many cell types other than reactive astrocytes during ND. NF-κB is activated in dopaminergic neurons in the SNpc of PD patients (Hunot et al., 1997), in peripheral immune cells in patients with HD (Trager et al., 2014) and, in hippocampal and entorhinal cortex neurons of AD patients, but not in glial cells (Terai et al., 1996; Kaltschmidt et al., 1997; Ferrer et al., 1998). Overall, this ubiquitous cascade is found activated in various cell types including astrocytes but these observations do not prove that NF-κB is required for astrocyte reactivity. In a mouse model of ALS, Crosio et al. found that inhibiting NF-κB signaling selectively in astrocytes only transiently attenuated their reactivity at the onset of disease (Crosio et al., 2011).

Overall, the NF-κB pathway is activated in ND and plays a key role in microglial activation, but this cascade does not seem essential to initiate astrocyte reactivity. Further studies in other models are needed to understand the role of NF-κB pathway in astrocyte reactivity during ND.

### The phosphatase calcineurin

The Ca^2+^/calmodulin-dependent serine/threonine phosphatase CN regulates gene expression by modulating transcription factors such as nuclear factor of activated T-cells (NFATs) and NF-κB (Furman and Norris, 2014). CN is a ubiquitous protein, although it is expressed at high levels in the brain. It regulates growth, differentiation and various cellular processes in T-cells, osteoclasts and myocytes (Hogan et al., 2003 and see Figure 1 for a detailed description of the pathway).

CN is activated in inflammatory conditions. Several studies have linked the CN/NFAT pathway to astrocyte reactivity, in particular in AD. Indeed, CN immunoreactivity is high in reactive astrocytes in aged mice and around amyloid plaques both in AD patients and mouse models (Furman and Norris, 2014). Activated NFAT1 and 3, two downstream targets of CN, are found in both neurons and astrocytes in AD brains (Abdul et al., 2009).

The effects of CN on astrocyte reactivity are extremely complex and context-dependent because CN can both trigger and prevent reactivity (Furman and Norris, 2014). On the one hand, overexpression of constitutively active CN (caCN) in primary rat hippocampal astrocytes induces morphological and transcriptional changes reminiscent of astrocyte reactivity *in vivo* (Norris et al., 2005). Viral-mediated overexpression of VIVIT, a blocking peptide that inhibits NFAT, attenuates astrocyte reactivity around amyloid depositions in APP/PS1dE9 mice (Furman et al., 2012). But on the other hand, caCN expression in astrocytes reduces GFAP induction following brain injury or LPS injection (Fernandez et al., 2007), and in APP/PS1 mice (Fernandez et al., 2012). This discrepancy between the pro- and anti-reactivity action of CN may be controlled by its signaling partners (Fernandez et al., 2012; Furman and Norris, 2014): its downstream targets (NF-κB vs. NFAT for example) as well as its activators. Indeed, Aβ and IGF-1 both activate CN in cultured astrocytes, but they have opposite effects on the NF-κB pathway (Pons and Torres-Aleman, 2000; Lim et al., 2013).

Overall, CN appears to modulate rather than induce astrocyte reactivity. Whether the effects of CN are conserved in other models and ND remains to be assessed.

### The MAPK pathway

The binding of growth factors (such as FGF, EGF, and TGFα), cytokines or extracellular matrix proteins to their specific cell-surface receptors activates the MAPK pathway (Jeffrey et al., 2007). This is mediated by the activation of small GTP-ase proteins (RAS) and the successive phosphorylation of MAP3K, MAP2K, and MAPK. There are three main phosphorylation cascades, with p38, ERK1/2 or JNK as downstream effectors (see Figure 1 for a detailed description of the pathway). All result in the activation of different transcription factors by phosphorylation. Cellular stress and extracellular matrix proteins such as integrins activate the c-jun N-terminal kinase (JNK) cascade, whereas cytokines such as IL-1β activate p38 (Jeffrey et al., 2007).

The MAPK pathway is activated in many cell types in ND patients and models. Reactive astrocytes contain active forms of p38, JNK and ERK in mouse models and/or in patients with ALS (Migheli et al., 1997; Tortarolo et al., 2003; Bendotti et al., 2004; Chung et al., 2005). However, in ALS mice, p38 is also activated in motor neurons and microglial cells (Tortarolo et al., 2003). Similarly, p38 and JNK are activated both in neurons and reactive astrocytes in the brain of patients with various tauopathies (Ferrer et al., 2001). In AD patients, phosphorylated forms of p38 are observed in neurons and glial cells around plaques (Hensley et al., 1999) but only in microglial cells in a mouse model (Koistinaho et al., 2002). Several MAPK inhibitors have been tested in an attempt to reduce neuroinflammation in pathological conditions; however, they are thought to act on reactive microglia (Kaminska et al., 2009).

Overall, although the MAPK pathway is activated in many cell types in ND patients and mouse models, to the best of our knowledge, there is no evidence showing that it is directly involved in the initiation of astrocyte reactivity in ND.

### Additional levels of complexity

#### Interactions between pathways

**Figure 3:**
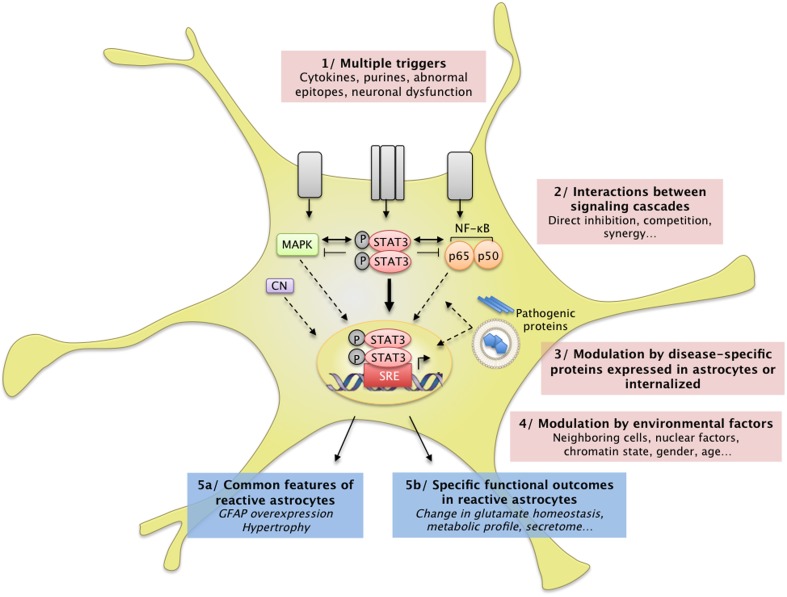
**Complex interactions between intracellular cascades result in a unique pattern of astrocyte reactivity in ND**. (1) Many signals can trigger astrocyte reactivity (see Section How do Astrocytes become Reactive? and Figure 1) such as cytokines, purines or abnormal epitopes like aggregated proteins. Astrocytes may also react to the absence of “resting signals” from neighboring cells, which occurs with neuronal death in ND. (2) These molecular signals are detected by specific receptor complexes at the astrocyte membrane, which activate several signaling cascades such as the JAK/STAT3 pathway, the NF-κB pathway, and the MAP kinase pathway (see also Figure 1). These pathways and their effectors interact either directly or indirectly, in the cytoplasm, in the nucleus, or on DNA promoter regions (see Section Additional Levels of Complexity). Recent studies support the idea that these cascades eventually converge on the JAK/STAT3 pathway, triggering a transcriptional program of reactivity in astrocytes. This program is modulated at several levels. (3) Disease-specific proteins (mHtt, mSOD1) endogenously expressed in astrocytes as well as internalized aggregated proteins (Aβ) can directly interfere with these signaling cascades or with transcriptional activity. (4) Several environmental factors may also affect the transcriptional program in reactive astrocytes, such as nuclear factors (other transcription factors, chromatin state) and environmental factors (age, sex). In the brain, dialog with other cell types or the specific brain regions involved is another potential level of modulation. These complex signaling cascades result in (5a) common features of astrocyte reactivity such as the upregulation of GFAP and cellular hypertrophy and (5b) disease-specific outcomes. This scheme illustrates how several signals can converge on a central signaling cascade that, in turn, is modulated in a disease- and environment-specific manner to produce a particular functional outcome.

There are many levels of crosstalk between these intracellular signaling pathways (Figures 1, 3). For instance, depending on the specific cellular environment, STAT3 and NF-κB pathways may interfere with each other through direct physical interaction, perform reciprocal inhibition through their respective inhibitors, or cooperate in the regulation of transcription of target genes (Grivennikov and Karin, 2010; Oeckinghaus et al., 2011). Similar interactions between STAT3 and ERK have been reported *in vitro* (Jain et al., 1998). In astrocyte cultures, the stimulation of purinergic receptors by specific agonists results in STAT3 phosphorylation, suggesting crosstalk between STAT3 and purinergic signaling cascades (Washburn and Neary, 2006). Similarly, several members of the MAPK family may interact with the NF-κB pathway. For example, p38 is a co-factor for NF-κB activation (Hoesel and Schmid, 2013). However, most of these mechanisms were described in cell lines, using cytokine stimulation or expression of constitutively active mutant proteins. Whether these interactions occur in reactive astrocytes *in vivo* remains to be demonstrated, especially in ND.

In addition, transcription factors such as STAT3 can also bind non-consensus sequences and interact with co-factors or epigenetic regulators, which represent an additional level of transcriptional regulation (Hutchins et al., 2013). Zamanian et al. recently showed that astrocyte reactivity induced by LPS injection or ischemia in the mouse brain induces the expression of hundreds of genes (Zamanian et al., 2012). Only a subset of these genes was common between the two models, illustrating the diversity of the transcriptional changes that may occur in reactive astrocytes, depending on the trigger and cellular environment (see Figure 3).

#### MicroRNA

MicroRNAs (miRNA) are non-coding RNA involved in the post-transcriptional regulation of gene expression. Several studies have linked changes in miRNA expression to astrocyte reactivity (Bhalala et al., 2013). For example, the expression of particular miRNA increases in parallel with markers of reactivity, in the brains of patients and models of AD (Li et al., 2011) and in mouse astrocytes after spinal cord injury (Bhalala et al., 2012). Furthermore, miR145 reduces GFAP expression (Wang et al., 2015), whereas miR181 controls the expression of several cytokines in astrocytes (Hutchison et al., 2013). These miRNAs can also modulate signaling cascades including the NF-κB (Cui et al., 2010) and JAK/STAT pathways (Witte and Muljo, 2014). Overall, miRNAs, by regulating gene networks, add another level of control to astrocyte reactivity, which requires further investigation in ND.

#### The effects of mutant proteins

Interestingly, mutant proteins involved in familial forms of ND may also interfere directly with intracellular cascades and thus affect signaling that reaches the nucleus (Figure 3). For example, wild-type Htt plays a role in NF-κB nuclear transport (Marcora and Kennedy, 2010) and mHtt impairs NF-κB signaling in astrocytes (Chou et al., 2008). In addition, mHtt interacts with the inhibitor of κB kinase (IKK) and inhibits IKK activity and NF-κB signaling (Khoshnan et al., 2004). Finally, ND are associated with dysfunction of the ubiquitin proteasome system (UPS, see Section Processing of Mutant Proteins), which is responsible for the degradation of IκB, the master inhibitor of NF-κB (Oeckinghaus et al., 2011, Figure 1). JAK proteins can also be targeted to the UPS by SOCS3 (Kershaw et al., 2014). Alterations in the UPS may thus indirectly influence the activity of several signaling cascades within astrocytes.

In conclusion, astrocyte reactivity can be triggered by many extracellular or intracellular signals. Although several signaling pathways are activated in reactive astrocytes, they seem to converge on the JAK/STAT3 pathway (Figure 3). Other cascades such as the NF-kB pathway or CN may regulate, rather than induce, astrocyte reactivity in ND. These signaling pathways result in massive transcriptional changes that may affect many astrocyte functions.

## What do reactive astrocytes do or fail to do during ND?

### Insights from cytokine-induced astrocyte reactivity

As a first attempt to elucidate the functional changes occurring in reactive astrocytes, cytokines and pro-inflammatory agents were overexpressed directly in the brain to induce reactivity. This was achieved by the injection of recombinant proteins, viral vector-mediated gene transfer or through transgenic mice overexpressing the protein of interest. Clearly, many functional changes are triggered by exposure to these molecules (Sofroniew, 2014). In addition to astrocytes, microglial cells also become activated and peripheral immune cells can be recruited within the brain parenchyma. Interestingly, reactivity can be selectively induced in astrocytes, leaving microglial cells virtually unaffected, by overexpressing the cytokine CNTF (Lavisse et al., 2012), or through the genetic ablation of β1-integrin in astrocytes (Robel et al., 2009). Such approaches have contributed to identify functional changes occurring in reactive astrocytes, including changes in glutamate homeostasis (Escartin et al., 2006; Beurrier et al., 2010), energy metabolism (Escartin et al., 2007; Carrillo-De Sauvage et al., 2015) and K^+^ homeostasis (Seidel et al., 2014; Robel et al., 2015); see Liberto et al. (2004) for a general review.

More relevant to ND, experiments have also been performed with disease-causing agents like Aβ or mHtt to decipher the functional changes occurring in reactive astrocytes during ND.

### Insights from disease models

In the following paragraphs, we will present several astrocyte functions that are known to be modified by reactivity, instead of describing each ND separately, to illustrate the existence of shared mechanisms between several ND.

#### Glutamate homeostasis

Alteration of glutamate uptake is probably one of the best documented and earliest described dysfunction of astrocytes in ND (Maragakis and Rothstein, 2004; Soni et al., 2014). Indeed, astrocytes are responsible for most glutamate uptake at synapses, through transporters encoded by *excitatory amino acid transporter gene 1 and 2* (EAAT1 and 2, also called GLAST and GLT1 in rodents) (Danbolt, 2001). Inefficient glutamate uptake leads to over-stimulation of glutamate receptors, which causes excitotoxic cell death in neurons. Excitotoxicity is a well-described pathological mechanism in ND (Maragakis and Rothstein, 2004).

Pioneering work from the Rothstein laboratory showed that glutamate transport is impaired in synaptosomes from patients with ALS (Rothstein et al., 1992). EAAT2 protein levels are low in the spinal cord and motor cortex of patients with familial or sporadic ALS (Rothstein et al., 1995) and animal models expressing mSOD1 (Bendotti et al., 2001; Howland et al., 2002), even before neuronal loss (Howland et al., 2002).

EAAT2 mRNA (Arzberger et al., 1997) and protein (Faideau et al., 2010) levels are also decreased in the caudate and putamen of patients with HD, depending on the disease stage (Faideau et al., 2010). In the prefrontal cortex of patients with HD, the uptake of glutamate is significantly impaired (Hassel et al., 2008). Such alterations are reproduced in mouse and fly models of HD (Lievens et al., 2001, 2005). Importantly, selective expression of mHtt in striatal astrocytes is sufficient to reduce GLT-1 expression, alters glutamate uptake and is associated with the dysfunction of striatal neurons (Faideau et al., 2010) and motor abnormalities (Bradford et al., 2009). Thus, alteration of glutamate uptake in astrocytes contributes to the neuronal toxicity observed in HD.

Alteration of glutamate homeostasis is also thought to contribute to the pathogenesis of AD. Binding of [^3^H] aspartate (a transportable analog of glutamate that does not bind to glutamate receptors) is reduced in the midfrontal cortex of patients with AD (Masliah et al., 1996). In a transgenic mouse model of AD, aspartate binding and glutamate transporter levels are lower than in WT littermates (Masliah et al., 2000). However, mRNA levels of glutamate transporters were unaffected in this model, suggesting that post-transcriptional modifications are involved. Indeed, in protein lysates from AD brains and Aβ-treated synaptosomes, EAAT2 is oxidized, which may impair its function (Lauderback et al., 2001). In addition, alternative EAAT2 splice variants with reduced glutamate transport capability (Scott et al., 2011) and abnormal detergent-insoluble EAAT2 (Woltjer et al., 2010) are found in vulnerable brain regions of AD patients. Finally, in brain slices, Aβ_1–42_ treatment results in the internalization of GLT-1, which reduces glutamate clearance by astrocytes (Scimemi et al., 2013). Therefore, transcriptional, post-transcriptional and post-translational mechanisms account for the dysregulation of EAATs in AD.

The low expression and poor functionality of EAATs appears to be a truly universal feature of ND. Expression of the human tau protein under the GFAP promoter decreases EAAT expression in the brainstem and the spinal cord and impairs glutamate transport in synaptosomal preparations from the spinal cord (Dabir et al., 2006). The selective expression of α-synuclein^A53T^ in astrocytes also reduces GLT-1 levels in both pre-symptomatic and symptomatic mice and triggers the death of dopaminergic neurons in the SNpc (Gu et al., 2010).

Once taken up from the synaptic cleft by astrocytes, glutamate is metabolized into glutamine by GS. Glutamine is then transported back to neurons and used for the production of glutamate and GABA (Danbolt, 2001). GS expression is low in the temporal cortex of patients with AD (Le Prince et al., 1995) and in the hippocampus of 3xTg-AD mice (Olabarria et al., 2011). GS mRNA levels are also lower in R6/2 mice (Lievens et al., 2001) and in BACHD mice (Boussicault et al., 2014) than in WT littermates. In the mouse hippocampus, the reduction in GS expression in reactive astrocytes triggers GABA depletion and neuronal hyper-excitability (Ortinski et al., 2010). Therefore, alterations of the glutamate-glutamine cycle may directly contribute to neuronal dysfunction in ND.

Altogether, it is well established that glutamate homeostasis is altered in ND, both in patients and animal models. In addition to its action on synaptic receptors, glutamate also serves as a metabolic signal to promote glucose uptake (Pellerin and Magistretti, 1994). Therefore, any alteration of glutamate homeostasis is likely to affect brain energy metabolism.

#### Energy metabolism

Astrocytes are involved in complex metabolic interactions with neurons (Allaman et al., 2011). Their strategic location at the interface between intracerebral blood vessels and synapses make them ideally positioned to deliver neurons with blood-borne metabolic substrates, according to their energy needs (see Belanger et al., 2011, for review). It is still unclear how the morphological changes associated with reactivity affect blood vessels coverage and metabolite uptake by astrocytes. Several metabolic pathways such as the glutamate-glutamine cycle, cholesterol metabolism and glutathione production, are compartmentalized between neurons and astrocytes. This confers astrocytes with a pivotal regulatory role. Energy deficits are a common hallmark of various ND (Lin and Beal, 2006), suggesting that some metabolic interactions are altered when astrocytes become reactive in ND.

##### Glucose metabolism

Glucose is by far the preferred energy substrate for the brain. According to the astrocyte-to-neuron lactate shuttle hypothesis, in conditions of increased neuronal activity, more glucose is taken up by astrocytes and oxidized through glycolysis and redistributed to neurons in the form of lactate (Pellerin and Magistretti, 1994). Positron emission tomography (PET) imaging shows that cerebral glucose metabolism is impaired in HD and AD patients (Grafton et al., 1992; Fukuyama et al., 1994). However, it is not known whether such metabolic deficits originate from reactive astrocytes.

A comprehensive autoradiography analysis suggests that regional glucose metabolism is reorganized in aged BACHD mice (decreased glucose uptake in the striatum, increased in the hypothalamus). Neuron-astrocyte insert co-cultures were performed to identify the cellular origin of such deficits. They showed that expression of mHtt in astrocytes does not affect their own rate of glucose uptake. However, it impairs glucose uptake in neurons, regardless of their genotype, suggesting that HD astrocytes indirectly regulate neuronal metabolism by diffusible factors (Boussicault et al., 2014). In ALS, lactate was identified as one of the molecules that are released differently when astrocytes are reactive. SOD1^G93A^ mice express lower levels of the astrocytic lactate transporter Slc16a4 than WT mice, which reduces lactate release in the spinal cord. Decreased lactate production is also observed in spinal astrocytes from familial ALS patients (Ferraiuolo et al., 2011), and may be deleterious to neurons relying on this metabolic supply.

Astrocyte metabolism can be studied by NMR on brain extracts after i.p. injection of ^13^C-labeled acetate in animal models because this compound is metabolized preferentially by astrocytes (see Section Reactive Astrocytes as Biomarkers). Astrocytic “hyper-metabolism,” characterized by high incorporation of ^13^C into metabolic intermediates is observed in the brain of 7-month old 3xTg-AD mice (Sancheti et al., 2014) and in the cortex of a mouse model of a tauopathy (Nilsen et al., 2013). But opposite changes are observed in the frontal cortex in a rat model of AD (McGill-R-thy1-APP) (Nilsen et al., 2014). Also in favor of a decreased metabolic activity in reactive astrocytes, the transfer of glutamine to glutamate is reduced in 3xTg-AD mice (Sancheti et al., 2014). Similar conflicting effects of Aβ on astrocyte oxidative metabolism were reported in culture (see Allaman et al., 2010, and references therein). Overall, the metabolic changes occurring in reactive astrocytes during ND are quite contrasted, depending on the ND, the animal model and the stage of the disease considered.

Besides glucose uptake, many studies report mitochondrial dysfunction in ND (Lin and Beal, 2006). Some studies suggest that not only neurons display mitochondrial failure in ND, but reactive astrocytes too. Motori et al. performed an elegant imaging study of reactive astrocytes following stab wound injury. They reported that mitochondria undergo more fission events in reactive than in resting astrocytes (Motori et al., 2013). The exposure of pro-inflammatory cytokines had the same effect on mitochondria *in vitro* and resulted in impaired respiratory activity and reactive oxygen species (ROS) production (Motori et al., 2013). Transcriptomic analysis of astrocytes from AD patients indicates that mitochondrial genes, such as those involved in tricarboxylic acid cycle, are expressed at lower levels than in astrocytes of age-matched control individuals (Sekar et al., 2015). The exposure of astrocytes to Aβ reduces their mitochondrial membrane potential, which activates toxic enzymes such as poly (ADP-ribose) polymerase 1 and nicotinamide adenine dinucleotide phosphate (NADPH) oxidase, a potent pro-oxidant enzyme (Abeti et al., 2011). Similarly, mitochondrial respiration is altered in astrocytes isolated from the spinal cord of ALS rats, probably because of increased oxidative stress (Cassina et al., 2008). Therefore, although transient beneficial changes occur in reactive astrocytes during ND, overall, they display altered metabolism that may result in ROS production (see Section Antioxidants and ROS).

##### Cholesterol metabolism

Cholesterol, the most common steroid in humans, is a structural component of cell membranes and a precursor of steroid hormones. It also contributes to synapse formation and neuronal activity; therefore, defects in cholesterol homeostasis may have severe consequences on brain function (Pfrieger and Ungerer, 2011). Cholesterol synthesis and degradation are highly compartmentalized in astrocytes and neurons, respectively (Pfrieger and Ungerer, 2011). In particular, astrocytes express high levels of apolipoprotein E (ApoE) that carries cholesterol to neurons (Bu, 2009).

The ε4 allele of the *ApoE* gene is the major risk factor for sporadic AD. It increases the probability of developing AD by a factor of 3–4 (Corder et al., 1993). ApoE influences the pathogenesis of AD at multiple levels, by regulating cholesterol metabolism, APP processing and Aβ clearance (Bu, 2009). ApoE is a chaperone for the binding of Aβ to the low density lipoprotein receptor or low density lipoprotein receptor-related protein 1 on astrocytes (Koistinaho et al., 2004). This is an important route for Aβ clearance (see Section Processing of Mutant Proteins). In astrocytes from aged APP/PS1dE9 mice, there is a widespread reduction in the expression of enzymes and transporters linked to cholesterol metabolism including ApoE, suggesting a decrease capacity to clear Aβ in these mice (Orre et al., 2014).

Cholesterol biosynthesis is low in the brain of several mouse models of HD (Valenza et al., 2010). In primary astrocytes from HD mice, mRNA levels of genes for cholesterol biogenesis and efflux are substantially lower than in control astrocytes. In addition, lower amounts of ApoE are secreted by HD *in vitro* and it forms smaller lipoprotein particles in the cerebrospinal fluid of HD mice (Valenza et al., 2010). The impairment in cholesterol biosynthesis correlates with the number of CAG repeats, the amount of mHtt (Leoni and Caccia, 2014) and is eventually toxic to HD neurons (Valenza et al., 2015).

##### Connexin-based networks of astrocytes

Astrocytes form multicellular networks connected by their gap junctions composed of connexins. These networks are involved in K^+^ buffering but also deliver metabolic substrates to active synapses (Rouach et al., 2008).

**Figure 4:**
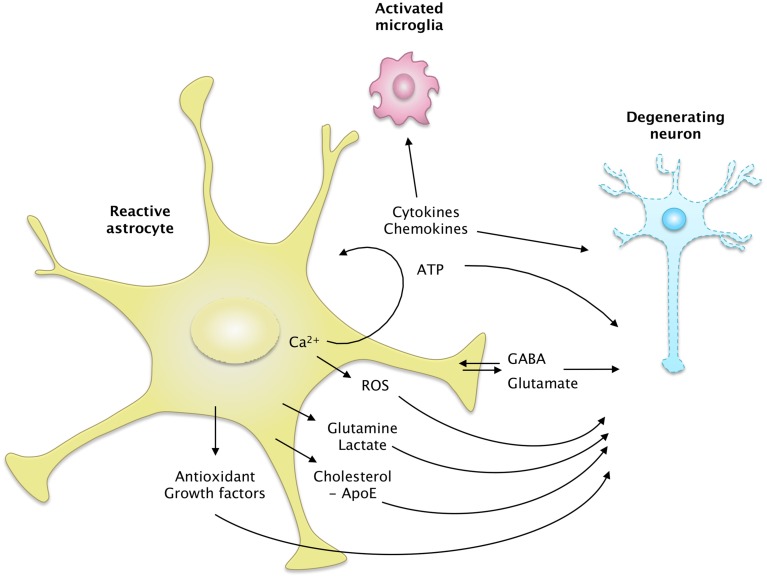
**The secretome of reactive astrocytes**. Astrocytes secrete many active molecules that influence neuronal survival and synaptic activity. Reactivity affects the pattern of secreted molecules, and thus alters neuron-astrocyte communications. In ND, reactive astrocytes may secrete higher levels of antioxidants, such as glutathione and its precursors or metabolic substrates. These changes would promote neuron survival. However, reactive astrocytes may also release fewer trophic molecules such as cholesterol, growth factors or glutamine and produce more ROS than resting astrocytes. The regulation of glutamate and GABA homeostasis may also be altered by reactivity, due to a change in their release but also their uptake. Intracellular Ca^2+^ levels are deregulated in ND, which may stimulate the release of gliotransmitters such as glutamate and ATP. Reactive astrocytes also produce more cytokines, which activate microglial cells or act as paracrine factors, maintaining glial cells in a chronically reactive state.

The expression of astrocyte connexin (Cx) and astroglial coupling through gap junction channels is changed in reactive astrocytes in ND (Giaume et al., 2010). Cx43 expression is high in the caudate of HD patients (Vis et al., 1998) and around amyloid plaques in the cortex of AD patients (Nagy et al., 1996). Similarly, Cx43 expression is higher in the spinal cord of SOD1^G93A^ mice (Cui et al., 2014), in two mouse models of AD (Mei et al., 2010) and in the MPTP model of PD (Rufer et al., 1996), than in their respective controls. Cx30 expression is also altered in ND models and patients, although the direction of the change is context dependent. Cx30 is expressed at low levels in the striatum of a rat and primate pharmacological model of PD (Charron et al., 2014) but is highly expressed in a mouse model of AD (Mei et al., 2010) and in AD patients (Nagy et al., 1996). However, in most cases, the functional effects on the astrocyte network and especially on metabolite trafficking was not assessed (Escartin and Rouach, 2013). Increased coupling may be beneficial for the delivery of metabolites. However, Cx also form hemichannels, through which several active molecules or gliotransmitters are released (Giaume et al., 2010; Bosch and Kielian, 2014). High Cx expression in reactive astrocytes in ND may thus lead to the excessive release of ATP or glutamate (Bosch and Kielian, 2014). This would maintain microglial cells in an active state and cause excitotoxicity in nearby neurons (see Section Release of Active Molecules and Figure 4).

#### Ion homeostasis

##### Buffering of K^+^

Astrocytes buffer K^+^ by specific channels and transporters, which are enriched in PAP and vascular endfeet. The maintenance of K^+^ homeostasis by astrocytes is essential for synaptic transmission and appears to be altered in ND. The mRNA expression of several K^+^ channels is lower in astrocytes isolated from APP/PS1dE9 than from WT mice (Orre et al., 2014). Protein levels of Kir4.1, an inward rectifier K^+^ channel, are decreased in the spinal cord of ALS mice (Kaiser et al., 2006) and in the striatum of R6/2 and zQ175 mice, two models of HD (Tong et al., 2014). Restoration of Kir4.1 levels through viral gene transfer in striatal astrocytes improves some of the neurological features in R6/2 mice (Tong et al., 2014). Importantly, astrocytes do not display the hallmarks of reactivity in these HD mice (see Table 1), suggesting that astrocytes can be dysfunctional before being “fully” reactive.

##### Ca^2+^ homeostasis

Several studies have reported alterations of Ca^2+^ homeostasis in reactive astrocytes in ND models, especially in AD (Vincent et al., 2010). Spontaneous Ca^2+^ transients are more frequent in slices from Tg2576 mice overexpressing APP than in controls (Pirttimaki et al., 2013). Hyperactive Ca^2+^ transients and waves can also be observed by two-photon live imaging with Ca^2+^ dyes in several mouse models of AD (Takano et al., 2007; Kuchibhotla et al., 2009; Delekate et al., 2014). Astrocytes from SOD1^G93A^ ALS mice also display enhanced Ca^2+^ transients following stimulation of mGluR5 receptors (Martorana et al., 2012) or exposure to ATP (Kawamata et al., 2014). Store-operated accumulation of Ca^2+^ in the endoplasmic reticulum may be responsible for these altered Ca^2+^ responses (Kawamata et al., 2014).

Deregulation of Ca^2+^ in reactive astrocytes may elicit profound changes in various Ca^2+^-dependent processes such as intracellular signaling cascades, proteolysis and gliotransmitter release.

#### Release of active molecules

Astrocytes interact with neighboring cells by releasing many molecules involved in cell-to-cell signaling, trophic support or antioxidant defense. The overall “secretome” of astrocytes is strongly altered by their reactivity (Figure 4). Some of these neuroactive molecules like glutamate, purines or GABA are neurotransmitters, and are thus called gliotransmitters when released by astrocytes (Araque et al., 2014).

##### Gliotransmitters

The release mode of gliotransmitters and their physiological relevance is a matter of intense debate (Araque et al., 2014; Sloan and Barres, 2014). The study of gliotransmission *in situ* is particularly difficult because most of these molecules are also released by neurons.

Glutamate is one of the best studied gliotransmitters. FRET-based imaging shows that cultured astrocytes release glutamate in response to recombinant Aβ1–42 or Aβ isolated from the brain of AD patients. This phenomenon is Ca^2+^-dependent and is deleterious to neighboring neurons (Talantova et al., 2013). Glutamate release elicited by mechanical stimulation is more important in cortical astrocytes isolated from BACHD mice than in their WT counterparts (Lee et al., 2013). Interestingly, the cytokine TNFα plays many regulatory roles at the excitatory synapse; it directly scales synaptic transmission and potentiates glutamate release by astrocytes (see Santello and Volterra, 2012, for review). TNFα levels are elevated in patients and animal models of ND (see Section Cytokines and Inflammatory Molecules), which may stimulate the non-physiological release of glutamate by reactive astrocytes. Unexpectedly, glutamate release in response to TNFα is impaired in hippocampal slices from AD mice harboring numerous Aβ plaques and reactive astrocytes (Rossi et al., 2005). The authors hypothesized that the intracellular cascades downstream from the TNFα receptor were altered in reactive astrocytes in this model (Rossi et al., 2005). Overall, additional studies are still needed, especially *in vivo*, to determine precisely how the release of glutamate by reactive astrocytes is changed in ND, and how it modulates synaptic transmission (Agulhon et al., 2012).

GABA is yet another gliotransmitter that was recently implicated in ND. Reactive astrocytes release more GABA than resting astrocytes, which contributes to cognitive impairment in two mouse models of AD (Jo et al., 2014; Wu et al., 2014). Excessive GABA released by reactive astrocytes results in the tonic inhibition of dentate gyrus granule cells in the hippocampus of AD mice. Inhibition of GABA synthesis or pharmacological blockade of GABA transporters restores synaptic plasticity and memory deficits in these mice (Jo et al., 2014; Wu et al., 2014). By contrast, GABA release by astrocytes appears to be defective in HD. Electrophysiological recordings on slices show that both GABA_A_ (postsynaptic) and GABA_B_ (presynaptic) currents are lower in R6/2 and zQ175 mice than in control mice. This results in a lower GABA-mediated tonic inhibition of striatal neurons. Pharmacological manipulation of the GABA transporter-3 (GAT-3), which is preferentially expressed by astrocytes, suggests that HD astrocytes have an impaired capacity to release GABA through GAT-3 (Wojtowicz et al., 2013).

Purines are another class of gliotransmitters, comprising ATP and its metabolite adenosine, which is generated extracellularly by ectonucleotidases. Stimulation of primary astrocyte cultures with Aβ induces the release of ATP (Jung et al., 2012) via Cx43 hemichannels (Orellana et al., 2011). Recently, it was shown that reactive astrocytes around amyloid plaques in the cortex of APPPS1 mice release more ATP via hemichannels than their WT counterparts. ATP, degraded into adenosine, acts as an autocrine signal on astrocyte P2Y1 receptors and elicits Ca^2+^ hyperactivity (Delekate et al., 2014). Cultured astrocytes from SOD^G93A^ mice also release more ATP than those from WT mice, which is toxic to co-cultured motor neurons (Kawamata et al., 2014).

##### Cytokines and inflammatory molecules

The levels of pro-inflammatory cytokines are higher in vulnerable brain regions and in the cerebrospinal fluid in ND patients than in healthy individuals (Lucin and Wyss-Coray, 2009; Heneka et al., 2014). However, many cell-types such as activated microglia or peripheral immune cells may produce these molecules. Transcriptional analysis performed on laser-captured GFAP^+^ reactive astrocytes from APP/PS1dE9 mice reveal that these cells express high levels of several cytokines (Orre et al., 2014). The number of genes induced and the fold-increase in expression are higher in astrocytes than in microglial cells, showing that reactive astrocytes may contribute significantly to the production of cytokines during AD. However, the absolute expression level of these cytokines remains lower in reactive astrocytes than in microglia. Some of these cytokines act as recruiting signals for peripheral immune cells or promote BBB permeability (Farina et al., 2007; Sofroniew, 2015). In ALS, a major increase in the transcription of inflammatory molecules is well established, including in astrocytes derived from both familial and sporadic forms of the disease (Haidet-Phillips et al., 2011). These astrocytes are toxic to motor neurons in co-culture systems.

In microglia, the maturation of some cytokines like IL-1β is operated in the cytosol by the inflammasome. Aβ phagocytosis activates the NOD-like receptor protein (NLRP) 3 inflammasome in these cells (Halle et al., 2008), thereby linking the internalization of pathologic proteins with the release of pro-inflammatory cytokines in ND. Only two recent studies suggest that in astrocytes, stimulation of the inflammasome also triggers IL-1β production (Minkiewicz et al., 2013; Zeis et al., 2015).

Finally, reactive astrocytes overexpress molecules of the complement system in AD mice (Orre et al., 2014), which can alter dendrite morphology, Ca^2+^ homeostasis and excitatory synaptic responses in neurons, at least *in vitro* (Lian et al., 2015). In AD and HD patients, components of the complement system are overexpressed (Singhrao et al., 1999; Lian et al., 2015); however they are not necessarily produced by astrocytes only.

##### Trophic factors

Astrocytes secrete various factors exhibiting trophic effects on neurons, such as growth factors (e.g., CNTF, brain-derived neurotrophic factor [BDNF], nerve growth factor [NGF], FGF), neurosteroids, and adhesion molecules involved in neurite outgrowth (Muller et al., 1995; Sofroniew and Vinters, 2010). Inadequate synthesis and release of such factors may contribute to neuronal toxicity observed in HD. The expression of mHtt in primary cultures of cortical astrocytes impairs BDNF production in astrocytes. Levels of mature BDNF in the medium are thus low under these conditions, which limits neurite development of primary cortical neurons (Wang et al., 2012). Similarly, transcription and release of the chemokine (C-C motif) ligand 5 (CCL5/RANTES), which promotes neurite outgrowth and neuronal survival, is also impaired by the expression of mHtt in cultured astrocytes (Chou et al., 2008). CCL5/RANTES accumulates in the cytosol of astrocytes in HD patients and in two mouse models of HD (Chou et al., 2008). Although reactive astrocytes secrete more trophic factors such as NGF in ALS rodent models and patients (Pehar et al., 2004; Ferraiuolo et al., 2011), it may nonetheless have unexpected detrimental consequences on nearby neurons. Indeed, vulnerable motor neurons in ALS express the specific p75 neurotrophin receptor isoform, and its stimulation by NGF triggers apoptosis instead of trophic actions (Pehar et al., 2004).

##### Antioxidants and ROS

Astrocytes are important for defense against ROS because they express many detoxifying enzymes and transporters (Vargas and Johnson, 2009; Allaman et al., 2011). They produce high levels of antioxidants for neurons, including ascorbic acid (AA, also known as vitamin C), glutathione and its precursors. The antioxidant action of astrocytes is crucial for neurons, because oxidative respiration produces high levels of ROS. Indeed, oxidative stress contributes to neuronal dysfunction in several ND (Belanger et al., 2011). The expression of many detoxifying enzymes and transporters are controlled by the master regulator NF-E2 related factor-2 (Nrf2), a transcription factor which translocates to the nucleus and binds specific promoter sequences in response to oxidative stress (Vargas and Johnson, 2009). Reactive astrocytes found in the spinal cord of early symptomatic ALS rats show high levels of Nrf2 expression and nuclear translocation (Vargas et al., 2005). Although high Nrf2 activity may be beneficial for neurons exposed to oxidative stress, this endogenous antioxidant response does not offer sufficient protection. Indeed, Nrf2 activity can be further enhanced in astrocytes by genetic manipulation, which improves disease outcome in animal models of ALS (Vargas et al., 2008), PD (Chen et al., 2009; Gan et al., 2012), and HD (Calkins et al., 2010).

The release of AA by astrocytes is altered in HD (Rebec, 2013). In the R6/2 mouse model of HD, extracellular AA levels are lower than in age-matched WT mice but only during behavioral activity (Rebec et al., 2002). Accordingly, mHtt expression in astrocytes (and not in neurons) is sufficient to trigger oxidative stress in neurons by diffusible factors (Boussicault et al., 2014). In fact, reactive astrocytes may not only produce fewer antioxidant molecules during ND, they may also release more pro-oxidant factors. Exposure to Aβ stimulates the pentose phosphate pathway in astrocytes *in vitro*; yet they release more ROS than in control conditions and are toxic to co-cultured neurons even without physical contact (Allaman et al., 2010). Furthermore, reactive astrocytes overexpress inducible NO synthase (NOS), including in the brain of AD patients (Heneka et al., 2014). Aβ peptides also cause a loss of mitochondrial membrane potential in astrocytes, which is associated with the activation of NADPH oxidase and excessive ROS production (Abramov et al., 2004). Accordingly, mitochondria from mSOD1 astrocytes produce large amounts of superoxide radicals, causing motor neuron death in co-culture, which is prevented by pre-incubation with antioxidants and NOS inhibitors (Cassina et al., 2008). Similarly, rodent astrocytes expressing a mutant form of TDP43 induce nitrosative stress in motoneurons and kill them (Rojas et al., 2014). Overall, ROS production by reactive astrocytes exposed to toxic or mutant disease-specific proteins seems to be another deleterious mechanism common to several ND.

#### Processing of mutant proteins

Aggregation of intra- or extra-cellular misfolded proteins is a central feature of ND. However, the exact role of aggregate formation is still debated. Soluble forms of mutant proteins are now considered to be the most toxic forms, and their aggregation may be instead a protective mechanism that prevents them from interfering with important intracellular partners (Ross and Poirier, 2004).

Misfolded proteins are degraded by two major intracellular pathways: autophagy and the UPS. Autophagy involves the formation of intra-cytoplasmic vesicles that may also envelop organelles. Engulfed elements are completely degraded by proteases such as cathepsins after fusion with a lysosome. Alternatively, the UPS forms a protease complex to which proteins are addressed by specific ubiquitin tags. Both pathways are altered in ND (Dantuma and Bott, 2014; Ghavami et al., 2014).

The UPS has been extensively studied in neurons in models of ND and is even a target of neuroprotection (Margulis and Finkbeiner, 2014; Popovic et al., 2014). Much less is known about the UPS in astrocytes (Jansen et al., 2014). Protein aggregates are mainly found in neurons, suggesting that astrocytes are more efficient than neurons at handling toxic proteins (Jansen et al., 2014). Indeed, a study based on a reporter system showed that the UPS is more active in glial cells than in neurons *in vitro* and *in vivo* (Tydlacka et al., 2008). During ND, the UPS in astrocytes may become less efficient than in healthy conditions because UPS subunits are down-regulated in astrocytes from AD patients (Simpson et al., 2011). In addition, during ND, reactive astrocytes may express a specific form of the proteasome, called the immunoproteasome, which is formed by the cytokine-inducible subunits β1i, β2i, and β5i. The immunoproteasome is detected in reactive astrocytes around amyloid plaques in AD patients and APP/PS1dE9 mice (Orre et al., 2013) and in the spinal cord of SOD^G93A^ mice (Puttaparthi and Elliott, 2005). The immunoproteasome is involved in antigen presentation (Jansen et al., 2014), but its functional role in reactive astrocytes during ND is not yet known. Invalidation of the B1i subunit of the immunoproteaseome does not influence disease outcome in SOD^G93A^ mice (Puttaparthi et al., 2007).

In AD, reactive astrocytes play yet another role in the clearance of extracellular Aβ. More than a decade ago, it was shown that astrocytes are able to internalize amyloid plaques and Aβ peptides (Funato et al., 1998; Nagele et al., 2003; Wyss-Coray et al., 2003). They do so by phagocytosis or by internalizing Aβ bound to membrane receptors, including ApoE receptors (Koistinaho et al., 2004; Thal, 2012, see Section Cholesterol Metabolism). The astrocytic protein ApoE also promotes Aβ extrusion through the BBB or along the perivascular space (Bu, 2009, and see Section Cholesterol Metabolism). Intracellular vesicles containing Aβ are addressed to lysosomes for degradation and the enhancement of lysosomal biogenesis selectively in astrocytes attenuates amyloid-related disease in a mouse model of AD (Xiao et al., 2014).

Aβ may also be degraded extracellularly, and astrocytes produce some Aβ-degrading enzymes such as insulin-degrading enzyme (IDE), neprilysin or matrix metalloproteinase 2 and 9 (MMP9). Neprilysin and IDE are overexpressed in reactive astrocytes in contact with plaques in AD brains (Apelt et al., 2003; Dorfman et al., 2010) and MMP9 is overexpressed in mouse models of AD (Yan et al., 2006). However, reactive astrocytes may eventually become overwhelmed as the disease progresses because they undergo cell lysis and form extracellular deposits containing neuronal-derived Aβ peptides (Nagele et al., 2003).

Alternatively, it was suggested that reactive astrocytes may contribute to Aβ production by overexpressing β-site APP cleaving enzyme 1 (BACE1), the rate limiting enzyme for Aβ production. Strong BACE1 expression is observed in reactive astrocytes in patients and several mouse models of AD, and following exposure to pro-inflammatory cytokines, which can directly activate the BACE1 promoter (Cole and Vassar, 2007). However, it is unknown how the amount of Aβ produced by astrocytes compares with the large pool of Aβ generated by neurons.

Regarding HD, we found that blocking astrocyte reactivity by overexpressing SOCS3 significantly promoted the formation of mHtt aggregates in the mouse striatum (Ben Haim et al., 2015). A recent study performed in *Drosophila* also reported that reactive glia are able to phagocyte mHtt expressed in neurons (Pearce et al., 2015). These results suggest that reactive astrocytes may participate in the processing of mHtt and its aggregation in neurons, but the exact molecular mechanisms need to be established.

## How do reactive astrocytes globally contribute to ND?

We have seen that many changes occur in astrocytes during ND, which makes it extremely difficult to get a clear view of their impact on the disease. In addition, the reactive status of astrocytes is not always directly reported in studies. Therefore, to evaluate the overall contribution of reactive astrocytes to ND, experimental designs that interfere with astrocyte reactivity provide a valuable insight into this difficult question (Table 3).

**Table 3:** **Main genetic approaches to block reactive astrocytes in mouse models of ND**.

**Approach**	**Construct**	**ND**	**ND model**	**Effect on astrocyte reactivity**	**Effects on disease outcomes**	**References**
Disruption of cytoskeleton in reactive astrocytes	gfap^−∕−^	ALS	SOD1^H46R^ mice	No change in vimentin protein levels No data on astrocyte morphology	Shorter lifespan No effect on motor symptoms	Yoshii et al., 2011
	gfap^−∕−^ vimentin^−∕−^	AD	APP/PS1dE9 mice	Lower astrocyte hypertrophy	Higher amyloid load More dystrophic neurites	Kraft et al., 2013
					No effect on amyloid load	Kamphuis et al., 2015
Ablation of proliferating astrocytes	gfap-tk	ALS	SOD1^G93A^	No change in the number of GFAP^+^ cells in the ventral SC No data on astrocyte morphology	No effect on survival, disease onset, duration No effect on motor function No effect on neuronal loss	Lepore et al., 2008a
Inhibition of the JAK/STAT3 pathway	lenti-socs3	HD	Lenti-Htt82Q	Lower GFAP and vimentin expression (mRNA and protein) Resting-like morphology	No effect on neuronal loss More mHtt aggregates	Ben Haim et al., 2015
Inhibition of the NF-kB pathway	hGFAP-Cre x IKKβ ^fl∕fl^	ALS	SOD1^G93A^ mice	No data on astrocyte phenotype	No effect on survival No effect on motor performances	Frakes et al., 2014
	AAV-IκBα-SR	ALS	SOD1^G93A^ mice	No data on astrocyte phenotype	No effect on survival No effect on motor performances No effect on neuron survival *in vitro*	Frakes et al., 2014
	hGFAP-IκBα-DR	ALS	SOD1^G93A^ mice	Temporary lower number of GFAP^+^ cells (at disease onset)	No effect on survival No effect on motor performances	Crosio et al., 2011
	lenti-DN-IKKγ	HD	R6/2 mice	No data on astrocyte phenotype	Improved motor and cognitive deficits, Less severe MSN atrophy	Hsiao et al., 2013
Inhibition of CN/NFAT signaling	AAV-VIVIT	AD	APP/PS1dE9	Trend of lower GFAP levels (protein) Reduced astrocyte hypertrophy	Improved cognitive deficits Improved synaptic transmission Lower amyloid load	Furman et al., 2012
Constitutive activation of CN	mGFAP-caCN	AD	APP/PS1	Fewer GFAP^+^ cells Lower GFAP levels (protein and mRNA) Reduced astrocyte hypertrophy around plaques	Reduced cognitive deficits Lower amyloid load	Fernandez et al., 2012

*Abbreviations: AAV, adeno-associated viral vector; caCN, constitutively active form of calcineurin; DN, dominant negative; IκBα, nuclear factor of kappa light polypeptide gene enhancer in B-cells inhibitor alpha; IKKβ, IκBα kinase alpha; MSN, medium-sized spiny neurons; SC, spinal cord; SR, super repressor; tk, thymidine kinase; DR, degradation resistant*.

### Intermediate filament KO

Given that the upregulation of IF is a hallmark of astrocyte reactivity, transgenic mice knockout (KO) for GFAP or Vim or double KO were initially generated and tested in acute injuries. Disruption of reactive astrocyte cytoskeleton was also studied in ND models and was found to shorten the lifespan of the SOD1^H46R^ mouse model of ALS (Yoshii et al., 2011). Studies involving the genetic ablation of GFAP and vimentin in APP/PS1dE9 mice gave conflicting results, with one reporting increased amyloid load and dystrophic neurites (Kraft et al., 2013), and another showing no effect on amyloid load in the cortex (Kamphuis et al., 2015). However, knocking out IF affects several basal functions in astrocytes (Shibuki et al., 1996) and results in many transcriptional changes (Kamphuis et al., 2015). Upregulation of IF is only a hallmark of reactivity; therefore, their removal from astrocytes will not necessarily block other molecular cascades associated with reactivity.

### Ablation of proliferative astrocytes

Another strategy developed to evaluate the contribution of reactive astrocytes to CNS injury is the ablation of proliferating astrocytes. This approach involves the expression of the viral enzyme thymidine kinase (TK) under the GFAP promoter, in presence of the drug ganciclovir (Bush et al., 1999). Ganciclovir is metabolized by TK-expressing cells into a base analog that blocks DNA replication, thus inducing the death of proliferating cells. This system has been extensively used to evaluate the effects of glial scar formation in acute injury models (Sofroniew, 2009). These studies demonstrate that glial scar-forming astrocytes act as a barrier to limit immune cell extravasation in the CNS parenchyma. However, in the progressive SOD1^G93A^ mouse model of ALS, ablation of proliferating astrocytes has no effect on disease outcomes (Lepore et al., 2008a), probably because of the small number of proliferating astrocytes in this model (see Section Do Reactive Astrocytes Proliferate in ND?).

### Manipulation of intracellular signaling pathways

A third strategy to study reactive astrocytes is to block intracellular signaling pathways controlling reactivity.

#### The JAK/STAT3 pathway

Several transgenic mice have been generated to block the JAK/STAT3 pathway in reactive astrocytes. They are based on the conditional KO of STAT3 following the expression of the Cre recombinase under the GFAP or nestin promoter (Okada et al., 2006; Herrmann et al., 2008). Most of these studies have focused on acute injuries with glial scar formation and found that reactive astrocytes mainly exert beneficial functions. By contrast, few studies have investigated the contribution of the JAK/STAT3 pathway in reactive astrocytes in ND, using pharmacological or viral-based approaches. In the MPTP mouse model of PD, pharmacological inhibition of JAK2 reduces astrocyte reactivity. However, this inhibitor does not influence tyrosine hydroxylase levels in the striatum, suggesting that reactive astrocytes do not contribute to dopaminergic loss in this model (Sriram et al., 2004). In a mouse model of HD, viral-mediated overexpression of SOCS3 in reactive astrocytes did not influence neuronal death but promoted the formation of mHtt aggregates (Ben Haim et al., 2015). This intriguing result suggests that reactive astrocytes affect the processing and aggregation of mHtt, which is key pathological mechanism in HD (see Section Processing of Mutant Proteins).

#### The NF-κB pathway

In ALS, two independent studies reported that inhibition of the NF-κB pathway in reactive astrocytes does not influence disease phenotype in SOD1^G93A^ mice (Crosio et al., 2011; Frakes et al., 2014). To block this pathway in astrocytes, Frakes et al. crossed mice KO for IKKβ in astrocytes (GFAP-Ikkb^flfl^) with SOD1^G93A^ mice or they overexpressed a dominant negative form of IκBα (AAV-IκB-SR) in astrocytes by viral gene transfer. The inhibition of the NF-κB pathway in reactive astrocytes did not influence motor neuron survival in culture or in the spinal cord of SOD1^G93A^ mice (Frakes et al., 2014), probably because this pathway is mainly active in microglia.

The role of the NF-κB pathway in reactive astrocytes has also been studied in HD. A dominant negative form of IKKγ (DN-IKKγ) was overexpressed by lentiviral gene transfer in the striatum of R6/2 mice to block NF-κB signaling. DN-IKKγ overexpression improved motor performance and prevented shrinkage of striatal neurons in HD mice (Hsiao et al., 2013). However, DN-IKKγ expression was not restricted to astrocytes and may thus have acted in other cell types such as microglia.

#### The CN/NFAT pathway

CN is activated upon inflammatory stimulation and regulates gene expression through the transcription factors NFATs and NF-κB (Furman and Norris, 2014). Expression of caCN in astrocytes of APP/PS1 mice reduces astrocyte reactivity, Aβ levels and the number of amyloid plaques. These effects are associated with improved cognitive functions (Fernandez et al., 2012). The beneficial effects of CN are mediated by the inhibition of the NF-κB pathway and subsequent production of pro-inflammatory cytokines (Fernandez et al., 2012). Viral-mediated gene transfer of the blocking peptide VIVIT was used to inhibit NFAT signaling in hippocampal astrocytes in APP/PS1dE9 mice (Furman et al., 2012). VIVIT limited astrocyte hypertrophy, prevented the accumulation of Aβ and improved synaptic plasticity and cognitive functions in AD mice (Furman et al., 2012). These results suggest that reactive astrocytes play detrimental roles in AD. However, VIVIT may be secreted by infected astrocytes; therefore, it is not possible to exclude the involvement of other cell types, especially because CN is permanently activated in neurons in the Tg2576 mouse model of AD (D'Amelio et al., 2011).

In conclusion, several approaches have been used to determine the contribution of reactive astrocytes to ND progression. The overall picture is still unclear because reactive astrocytes have been shown to be beneficial, detrimental or to have no effect, depending on the experimental approach chosen, the molecular target (e.g., IF, signaling cascades) and the disease model (Table 3). Some of these approaches rely on pharmacological inhibitors or transgenic mice lacking cell-type specificity or that might involve developmental effects (with non-inducible Cre expression for example). To better delineate the roles of reactive astrocytes in ND, it will be interesting to target pivotal signaling cascades and to use cell-type specific and versatile tools like viral vectors to interfere with astrocyte reactivity in different ND models.

## Ongoing questions, future directions

### Heterogeneity of reactive astrocytes

One of the next challenges in the field is to deal with the functional heterogeneity of astrocytes. Indeed, like neurons, astrocytes display remarkable heterogeneity regarding their density, morphology (Emsley and Macklis, 2006), transcriptional profile (Bachoo et al., 2004), and expression of transporters, channels, receptors and transcription factors (Matyash and Kettenmann, 2010). Astrocyte reactivity is also quite heterogeneous both between and within brain regions (Anderson et al., 2014). Such heterogeneity is best explored in acute injury models, because injury can be inflicted in different brain regions. In the spinal cord for example, astrocytes from the ventral horn do not migrate into a dorsal stab wound, even for very close lesions (Tsai et al., 2012). Even within the same sub-region of the mouse cerebral cortex, astrocytes respond heterogeneously to stab wound injury. A very elegant study based on live two-photon microscopy demonstrated that almost all astrocytes become hypertrophic and overexpress GFAP following injury; however, some had their processes polarized toward the lesion, others proliferated (less than 15%), and some remain static (Bardehle et al., 2013). In ND, reactive astrocytes in contact with plaques have a more pronounced reactive morphology than those at a distance, which correlates with larger transcriptional changes (Orre et al., 2014). In the 3xTg-AD model of AD, astrocytes at distance from plaques may even be atrophic (Olabarria et al., 2010). Overall, the heterogeneity of reactive astrocytes at the regional, sub-regional and cellular level needs to be thoroughly investigated in animal models and patients taking advantage of modern techniques such as two-photon microscopy and cell-specific transcriptomic analysis. Indeed, it remains unclear how such heterogeneity is established during development or disease and how it contributes to the local vulnerability of neighboring neurons in ND (Molofsky et al., 2012).

### Reactive astrocytes in the clinics

#### Reactive astrocytes as biomarkers

Given that astrocytes are able to sense even mild neuronal dysfunction and to become reactive, they represent attractive biomarkers for the diagnosis and monitoring of ND. Reactive astrocytes can be imaged in brain slices or in living mice by the expression of a reporter gene (GFP, luciferase) under the control of the GFAP promoter (see O'Brien et al., 2013, for a complete review). For clinical applications, non-invasive image techniques to monitor reactive astrocytes are still under development.

PET provides a way to quantify neuroinflammation through radiolabeled tracers that bind to glial cells. The most common target for neuroinflammation is the peripheral benzodiazepine receptor or translocator protein 18 kDa (TSPO) (see Chauveau et al., 2008). Activated microglia express high levels of TSPO but, as recently demonstrated, reactive astrocytes also overexpress this protein (Lavisse et al., 2012). Therefore, TSPO radioligands do not discriminate between reactive microglia and reactive astrocytes, but they are nonetheless a valuable imaging approach to identify early neuroinflammation in ND patients (Venneti et al., 2006). Radiotracers that target astrocyte metabolism, such as [1-^11^C]-octanoate (Kuge et al., 2000) and [2-^18^F]-fluoroacetate (Marik et al., 2009) have also been evaluated in models of glioblastoma and ischemia. It remains to be established whether they can detect progressive astrocyte reactivity in ND patients. Another molecular target is monoamine oxidase B (MAO-B), which is highly expressed in reactive astrocytes. The binding of ^11^C-DED, a MAO-B radioligand is high in patients with ALS (Johansson et al., 2007), and patients with mild cognitive impairment or AD (Carter et al., 2012). However, this enzyme is also found in serotonergic neurons, which could contribute to this signal. Overall, new PET radiotracers with higher specificity for reactive astrocytes are needed and recent transcriptomic studies on reactive astrocytes may help to identify new targets.

Nuclear magnetic resonance (NMR) techniques are an attractive alternative to monitor astrocyte reactivity *in situ*. Increased T1 relaxation time is observed by magnetic resonance imaging (MRI) in acute models of ischemia and excitotoxicity. Arundic acid, an inhibitor of astrocyte reactivity, normalizes it, but the molecular basis for such changes in NMR signals is unclear (Sibson et al., 2008). NMR-spectroscopy (MRS) allows the quantification of abundant brain metabolites, including myo-inositol, glutamine and choline which are enriched in glial cells. In a model of selective astrocyte reactivity in the rat brain, myo-inositol and choline levels are higher whereas glutamine levels are lower than in controls, suggesting that reactivity leads to the complex re-structuring of metabolic pathways (Carrillo-De Sauvage et al., 2015). High concentrations of myo-inositol are also commonly observed in ND models and patients, which correlates with neuroinflammation (Choi et al., 2007). However, the exact contribution of reactive astrocytes to these NMR signals is unknown because of the concomitant activation of microglial cells or other pathological events in ND.

More cellular selectivity may be achieved by MRS techniques after the infusion of ^13^C-labeled metabolic substrates such as glucose or acetate. Indeed, acetate is preferentially oxidized by astrocytes and its metabolic fate can be monitored by MRS (De Graaf et al., 2011). Furthermore, the rate of astrocytic tricarboxylic acid cycle and of the glutamate/glutamine cycle can be estimated by modeling (Lebon et al., 2002). ^13^C-acetate injection coupled with *ex vivo* MRS analysis was performed recently in several rodent models of AD (see Section Cholesterol Metabolism). ^13^C-MRS may be translated to the clinics although it remains quite an expensive and sophisticated approach (Ross et al., 2003).

#### Reactive astrocytes as therapeutic targets

The above-mentioned changes in reactive astrocytes make these cells alternative or complementary therapeutic targets to neurons for ND (Escartin and Bonvento, 2008). For example, strategies enhancing glutamate uptake in astrocytes may prevent excitotoxicity, which is common to all ND (Soni et al., 2014). High-throughput screening identified β-lactam antibiotics as potent inducers of glutamate uptake by astrocytes (Rothstein et al., 2005). The β-lactam antibiotic ceftriaxone is neuroprotective *in vitro* and *in vivo* in models of ALS (Rothstein et al., 2005) and HD (Miller et al., 2008). A phase I clinical trial with ceftriaxone in ALS patients gave promising results, but they were not confirmed in the phase II-III stage (Cudkowicz et al., 2014). Another astrocyte-based therapeutic strategy involves grafting astrocyte progenitors close to vulnerable neurons to provide them with global support (Lepore et al., 2008b). Interestingly, some pharmacological agents tested or used in clinics to target neurons may also affect astrocyte functions. Indeed, neurons and astrocytes share many membrane receptors, transporters and signaling pathways. For example, activators of the Nrf2 pathway like curcumin may enhance antioxidant defense in the brain by acting within astrocytes (Vargas and Johnson, 2009).

Therapeutic strategies tested so far for ND have largely focused on neurons and have been mostly unsuccessful to date (Huang and Mucke, 2012; Wild and Tabrizi, 2014). Only symptomatic treatments are offered to patients and their efficacy of some decreases with disease progression (e.g., acetylcholine esterase inhibitors for AD, L-DOPA supplementation for PD). No treatment truly prevents neurons from degenerating. In light of their many actions on neurons, strategies targeting reactive astrocytes may effectively sustain neuronal function and hence survival during ND. However, given the complex changes that occur in reactive astrocytes during ND, complete ablation of astrocyte reactivity may be counterproductive because these cells also display beneficial adaptative changes during disease. Identifying the complex interplay between shared intracellular pathways mediating reactivity and disease specific signals may enable the design of selective therapeutic cocktails to engage reactive astrocytes in protective actions (Figure 3).

## Conclusions

Overall, this review illustrates the multifaceted and complex roles of reactive astrocytes during ND. Astrocyte reactivity appears as a conserved response that is initially beneficial but is later corrupted by disease-specific alterations. Huge progress has been made recently as a result of the heightened interest in glial cells, and the development of innovative and cell type-specific approaches. However, these cells remain enigmatic, and many aspects of their physiology need to be clarified. Although the molecular pathways leading to astrocyte reactivity during ND have been described, it is crucial to elucidate what disease-, region- and environmental-specific mechanisms control the functional outcomes associated with astrocyte reactivity (Figure 3). In any case, considering reactive astrocytes as key partners in neuronal dialog during ND opens new avenues for neuroscience and biomedical research.

### Conflict of interest statement

The authors declare that the research was conducted in the absence of any commercial or financial relationships that could be construed as a potential conflict of interest.
